# Oral herbal medicine for treatment of postherpetic neuralgia: A protocol for systematic review and meta-analysis

**DOI:** 10.1097/MD.0000000000032484

**Published:** 2022-12-30

**Authors:** Hyoseung Jeon, Suji Lee, Sung-A Kim, Unhyung Lee, Seunghoon Lee

**Affiliations:** a Department of Clinical Korean Medicine, Graduate School, Kyung Hee University, Seoul, Republic of Korea; b Department of Acupuncture and Moxibustion Medicine, Kyung Hee University Medical Center, Seoul, Republic of Korea; c Department of Acupuncture and Moxibustion, College of Korean Medicine, Kyung Hee University, Seoul, Republic of Korea.

**Keywords:** herbal medicine, meta-analysis, postherpetic neuralgia, protocol, systematic review

## Abstract

Postherpetic neuralgia (PHN) is the most common complication of shingling. Herbal medicine for PHN is clinically used to improve chronic pain and other symptoms that reduce the quality of life (QoL), such as sleep disturbances and emotional problems. This study aims to describe methods for synthesizing evidence for the effectiveness and safety of herbal medicine for treating PHN.

**Methods:**

Randomized controlled trials using herbal medicine in patients with PHN will be included in this review. Electronic databases such as MEDLINE via PubMed, EMBASE, Cochrane Library, Cumulative Index to Nursing and Allied Health Literature, China National Knowledge Infrastructure, WanFang, KoreaMed, Research Information Service System, Korean Studies Information Service System, Database Periodical Information Academic, Oriental Medicine Advanced Searching Integrated System, and Korea Citation Index will be searched without language limitations from their inception to September 2022. Two authors will perform quality assessments independently using the Cochrane risk-of-bias tool. The primary outcome will be pain intensity, and the secondary outcomes will be global impression, anxiety, depression, sleep disturbance, QoL, and safety. All data from eligible studies will be synthesized for meta-analysis.

**Results:**

This study will be a systematic review and meta-analysis to evaluate the effectiveness and safety of oral herbal medicine for treatment of PHN.

**Conclusion:**

This study will provide evidence for herbal medicine as a potential treatment for PHN which is advantageous not only for patients but also for researchers and policymakers.

## 1. Introduction

Postherpetic neuralgia (PHN) is defined as intractable pain persisting for over 3 months after the onset of healing of herpes zoster.^[1]^ This pain is characterized by throbbing, aching, burning, or stinging sensation, depending on the innervation of the rash.^[2]^ Neuropathic pain, such as PHN, is often associated with other complications, such as functional loss, anxiety, depression, and sleep disturbances, reducing quality of life (QoL) more than nociceptive pain.^[3,4]^ PHN is difficult to treat because various symptoms are intertwined with severe pain. Approximately 20% of patients with PHN experience pain for more than 1 year after the rash, and approximately 10% for more than 4 years.^[5]^ According to a systematic review of epidemiology for PHN,^[6]^ the risk of progression to PHN in patients with herpes zoster varies from 5% to over 30%. The incidence of herpes zoster and PHN commonly increases with age. Elderly patients with PHN are at an increased risk of comorbidities such as sleep disturbance, depression, and anxiety. These pain-related comorbidities occur in chronic patients, complicating overall pain management and compromising prognosis.^[7]^

In general, treatment of PHN is important to reduce pain and minimize potential side effects.^[2]^ Pharmacological treatments are the first-line treatment for PHN to reduce persistent pain. Oral medications such as tricyclic antidepressants, serotonin-norepinephrine reuptake inhibitors, pregabalin, gabapentin, and tramadol, and topical medications such as capsaicin patches and lidocaine patches are usually administrated.^[8]^ However, the analgesic effect of these drugs is limited,^[9]^ as only approximately 50% of patients have a satisfactory analgesic effect.^[10]^ In addition, these drugs can cause several side effects, such as nausea, vomiting, fatigue, and dizziness.^[11]^ As these side effects are high in the elderly, it is necessary to use of drugs with caution and consider other therapeutic approaches in treating PHN.^[7]^

Traditional medicines, such as acupuncture, moxibustion, and herbal medicine, can be considered potential treatments for PHN. According to a review of traditional medicine for PHN,^[12]^ acupuncture and herbal medicine are most commonly used clinically to relieve pain and improve comorbidities. Although many systematic reviews^[13–15]^ have shown that acupuncture is effective for patients with PHN, there are few reviews on the effectiveness of herbal medicine for patients with PHN.

One systematic review^[16]^ of herbal medicine for neuropathic pain showed gross improvement compared with conventional medicine in patients with diabetic peripheral neuropathy but did not include randomized controlled trials (RCTs) for PHN. In another review,^[17]^ herbal medicine was expected to be effective for pain and depressive symptoms in patients with PHN. However, there are limitations in presenting sufficient evidence for clinical use due to methodological deficiencies and insufficient high-quality RCTs. Thus, this systematic review will be conducted to evaluate the effectiveness and safety of oral herbal medicine in patients with PHN by updating the current research progress.

## 2. Methods

### 2.1. Study registration

This systematic review and meta-analysis are reported in accordance with the preferred reporting items for systematic reviews and meta-analysis protocols.^[18]^ This study was registered with PROSPERO (CRD42022355315).

### 2.2. Inclusion criteria

#### 2.2.1. Types of studies.

RCTs without language limitations will be included. Qualitative studies, observational studies, and gray literature will be excluded.

#### 2.2.2. Types of participants.

According to the definition of PHN in the International Association for the Study of Pain,^[1]^ adult patients (≥18 years) who complain of pain lasting more than 3 months after the onset of healing of shingles will be included.

#### 2.2.3. Types of interventions.

Any oral herbal medicine administered for more than 2 weeks will be eligible, but patches, fumigation, and pharmacopuncture will be excluded. Studies with no treatment/waitlist group, placebo group, or a conventional treatment group as the control group will be included. In this review, conventional treatment refers to treatment recommended as level A or higher in the guidelines of the European Federation of the Neurological Societies,^[19]^ or as the first-line or second-line treatment in Neuropathic Pain Special Interest Group guideline.^[20]^ In addition, studies using oral herbal medicine as an add-on to conventional treatment or other complementary and alternative medicines will be included.

#### 2.2.4. Types of outcome measures.

##### 2.2.4.1. Primary outcomes.

The primary outcome measure will be pain intensity measured by a visual analog scale, numerical rating scale, or other validated scales to evaluate pain.

##### 2.2.4.2. Secondary outcomes.

As secondary outcome measures, the following scales will be evaluated:

- QoL assessed using a validated scale (e.g., 36-item Short-Form or Euro-QoL).
- clinical global impression of recovery.
- psychological measurements, such as depression or anxiety.
- sleep quality.
- fatigue.
- adverse events related to herbal medicine.

### 2.3. Search methods for identification of studies

#### 2.3.1. Electronic searches.

The following 12 databases will be searched from inception through September 30, 2022: 4 English databases (Medline via PubMED, EMBASE, Cochrane Library, and the Cumulative Index to Nursing and Allied Health Literature), 2 Chinese databases (China National Knowledge Infrastructure and WanFang), and 6 Korean databases (KoreaMed, Research Information Service System, Korean Studies Information Service System, Database Periodical Information Academic, Oriental Medicine Advanced Searching Integrated System, and Korea Citation Index).

#### 2.3.2. Search strategy.

The search terms will include PHN (herpes zoster, shingles, zoster, and neuralgia) and herbal medicine (traditional Chinese medicine, Chinese drugs, Kampo, Plants, and Herbs). The search strategies are presented online (see Table, http://links.lww.com/MD/I226, Supplemental Content, which illustrates the search strategy for Medline via PubMed, EMBASE, and the Cochrane library).

### 2.4. Data collection and analysis

#### 2.4.1. Selection of studies.

All retrieved studies will be imported into EndNote 20 software, and duplicated studies will be eliminated. Two authors (HJ and SAK) will independently screen studies by title and abstract, review the full text, and select the final eligible studies based on predefined criteria. Disagreements between the authors will be resolved by consensus through discussion or arbitration by the third author (SL).

#### 2.4.2. Data extraction and management.

Two reviewers (HJ and SAK) independently will extract the data using a predefined standard data extraction form. The data will include the basic characteristics of the study (first author, publication year, and country), characteristics of participants (sample size, average age, sex, and duration of disease), intervention (prescription name, duration of treatment, composition, dosage, formulation, and administration method), control type, and outcomes (pain intensity and secondary outcomes). Disagreements during the data extraction process will be resolved through discussions.

#### 2.4.3. Assessment of risk of bias.

The Cochrane Collaboration’s risk of bias tool will be used to independently assess the methodological quality of the included studies by 2 authors (HJ and SAK). The following domains will be assessed: random sequence generation, allocation concealment, blinding of participants and personnel, blinding of outcome assessments, incomplete outcome data, selective outcome reporting, and other sources of bias (baseline imbalance). Each domain is classified as low, unclear, or high risk of bias. If there is a disagreement between 2 authors, it will be resolved through discussion or consultation with the third author (SL).

#### 2.4.4. Measures of treatment effect.

The extracted data of treatment effects will be synthesized and analyzed using Review Manager software (RevMan version 5.4.1; the Nordic Cochrane Centre, Copenhagen, Denmark). The mean difference will be used to evaluate the treatment effect with 95% confidence interval (CI) for continuous data. A standardized mean difference will be used if the same outcome variable used different scales. The risk ratio will be used to evaluate the treatment effect with 95% CI for dichotomous data.

#### 2.4.5. Unit of analysis issues.

In the crossover design study, only the first-period data will be used to avoid carryover effects. Cluster RCTs will not be included in this review. When a trial will include more than 2 control groups within a meta-analysis, the data of the herbal medicine will be split equally and compared with each control group separately.

#### 2.4.6. Dealing with missing data.

For missing data, we will contact the corresponding author and request additional information for analysis. If the missing data cannot be retrieved, the analysis will be based on the available data.

#### 2.4.7. Assessment of heterogeneity.

Heterogeneity will be measured by visual inspection of the forest plots looking at the overlap of the CI. It will be identified by the *I*^2^ statistic and *χ*^2^ test with significance levels of *P* < .1, as recommended in the Cochrane handbook.^[21]^ The *I*^2^ statistic will be used to quantify the inconsistency among the included studies, with values interpreted as follows: unimportant heterogeneity (0%–40%), moderate heterogeneity (30%–60%), substantial heterogeneity (50%–90%), and considerable heterogeneity (75%–100%).^[21]^

#### 2.4.8. Assessment of reporting biases.

A funnel plot will be used to assess reporting bias if more than 10 trials are included.^[21]^

#### 2.4.9. Data synthesis.

RevMan version 5.4.1 software will be used to perform the meta-analysis. The pooled effect estimate will be calculated as a weighted average, and a random-effects model will be used to deal with potential heterogeneity among the included studies. If there is significant heterogeneity of included studies, quantitative analysis will not be performed.

#### 2.4.10. Subgroup analysis and investigation of heterogeneity.

If significant heterogeneity exists among the studies, subgroup analyses will be performed as follows:

- Duration of treatment (≥4 weeks or <4 weeks).
- Duration of the pain (≥6 months or <6 months).
- Types of herbal medicine formulae (classification according to pattern identification of Korean medicine such as blood stasis, qi deficiency, etc).

#### 2.4.11. Sensitivity analysis.

Sensitivity analysis will be performed to identify the robustness of our results based on methodological study quality (studies with low risk of allocation concealment) and sample size (studies with more than 40 participants in each group).

### 2.5. Ethics and dissemination

Written informed consent and ethical approval will not be required because this study is a systematic review. The main results of this review will be distributed through posters, newspapers, conference presentations, and peer-reviewed journals.

## 3. Discussion

PHN is a complex chronic disease (CCD) that requires attention because long-lasting pain results in poor QoL due to insomnia, fatigue, and emotional disorders.^[22,23]^ As conventional treatment strategies have limitations in treating CCDs,^[24]^ patients with long-term illnesses prefer alternatives to conventional medicine.^[25]^ Herbal medicine, in particular, is a complex ingredient that acts through various mechanisms and has therapeutic effects on CCD, in which various symptoms are complicatedly intertwined.^[26–28]^

A previous systematic review^[17]^ suggested that oral herbal medicines for PHN may be effective and safe for pain relief. However, these results should be interpreted with caution owing to methodological issues, the low quality of the included RCTs, or problems with the selection criteria used. In general, PHN is defined as pain lasting more than 3 months after the rash resolved;^[29–31]^ however, this review included studies that defined PHN as pain lasting 1 month. In addition, all the included studies were published before 2015, and it was necessary to analyze recently published RCTs. Therefore, this study aims to evaluate the evidence on the effectiveness and safety of herbal medicine for PHN by including the latest studies with appropriate inclusion criteria. These findings will guide doctors, healthcare policymakers, and patients regarding using herbal medicines for PHN and will be used as basic data for future clinical studies.

## Author contributions

**Conceptualization:** Hyoseung Jeon, Suji Lee, Seunghoon Lee.

**Data curation:** Hyoseung Jeon, Suji Lee, Sung-A Kim, Unhyung Lee.

**Formal analysis:** Hyoseung Jeon, Suji Lee, Sung-A Kim.

**Funding acquisition:** Seunghoon Lee.

**Project administration:** Hyoseung Jeon, Suji Lee, Sung-A Kim, Seunghoon Lee.

**Writing – original draft:** Hyoseung Jeon, Suji Lee.

**Writing – review and editing:** Suji Lee, Sung-A Kim, Unhyung Lee, Seunghoon Lee.

## Supplementary Material
