# Supplementary material for: Oral herbal medicine for treatment of postherpetic neuralgia: A protocol for systematic review and meta-analysis
Source: Medicine (Baltimore). 2022 Dec 30;101(52):e32484. doi: 10.1097/MD.0000000000032484 (PMC9803446; doi:10.1097/MD.0000000000032484)
Supplement: Supplementary file 1 [file medi-101-e32484-s001.pdf]

Supplemental Content _ Search strategy

1. Medline via PubMed

#1 neuralgia [Title/Abstract]

#2 "neuralgia, postherpetic" [Mesh Terms]

#3 PHN [Title/Abstract]

#4 ((postherpetic OR post-herpetic OR post herpetic) AND pain) [Title/Abstract]

#5 "Herpes Zoster"[Mesh Terms]

#6 zona [Title/Abstract]

#7 shingle* [Title/Abstract]

#8 zoster [Title/Abstract]

#9 “varicellovirus” [Mesh Terms]

#10 “herpesvirus 3, human” [Mesh Terms]

#11 HHV-3[Title/Abstract]

#12 #1 OR #2 OR #3 OR #4 OR #5 OR #6 OR #7 OR #8 OR #9 OR #10 OR #11

#13 “medicine, traditional” [Mesh Terms]

#14 alternative medicine* [Title/Abstract]

#15 complementary medicine* [Title/Abstract]

#16 “drugs, chinese herbal” [Mesh Terms]

#17 “plant extracts” [Mesh Terms]

#18 “plants, medicinal” [Mesh Terms]

#19 plant OR plants [Title/Abstract]

#20 “medicine, kampo” [Mesh Terms]

#21 (ethnopharmacology OR ethnomedicine OR ethnobotany) [Title/Abstract]

#22 (TCM OR T.C.M.) [Title/Abstract]

#23 herb* [Title/Abstract]

#24 "herbal medicine" [Mesh Terms]

#25 ((traditional OR chinese OR oriental OR herbal) AND medicine) [Title/Abstract]

#26 (phytodrug* OR phyto-drug* OR phytopharmaceutical*) [Title/Abstract]

#27 #13 OR #14 OR #15 OR #16 OR #17 OR #18 OR #19 OR #20 OR #21 OR #22 OR #23 OR #24 OR #25 OR #26

#28 #12 AND #27

2. EMBASE

#1 neuralgia:ab,ti

#2 'postherpetic neuralgia'/exp

#3 PHN:ab,ti

#4 (postherpetic OR ‘post-herpetic’ OR ‘post herpetic’) NEAR/2 pain

#5 'herpes zoster'/exp

#6 zona:ab,ti

#7 shingle*:ab,ti

#8 zoster:ab,ti

#9 'Varicellovirus'/exp

#10 'Varicella zoster virus'/exp

#11 ‘HHV-3’:ab,ti

#12 #1 OR #2 OR #3 OR #4 OR #5 OR #6 OR #7 OR #8 OR #9 OR #10 OR #11

#13 'traditional medicine'/exp

#14 ‘alternative medicine*’:ab,ti

#15 ‘complementary medicine*’:ab,ti

#16 'herbaceous agent'/exp

#17 (plant OR plants):ab,ti

#18 'plant extract'/exp

#19 'medicinal plant'/exp

#20 'Kampo medicine'/exp

#21 (ethnopharmacology OR ethnomedicine OR ethnobotany):ab,ti

#22 (TCM OR T.C.M.):ab,ti

#23 herb*:ab,ti

#24 (traditional OR chinese OR oriental OR herbal) NEAR/2 medicine

#25 (phytodrug* OR phyto-drug* OR phytopharmaceutical*):ab,ti

#26 #13 OR #14 OR #15 OR #16 OR #17 OR #18 OR #19 OR #20 OR #21 OR #22 OR #23 OR #24 OR #25

#27 #12 and #26

3. Cochrane library

#1 neuralgia:ti,ab

#2 MeSH descriptor: [Neuralgia, Postherpetic] explode all trees

#3 PHN:ti,ab

#4 (postherpetic OR post-herpetic OR post herpetic) near/2 pain:ti,ab

#5 MeSH descriptor: [Herpes Zoster] explode all trees

#6 zona:ti,ab

#7 shingle*:ti,ab

#8 zoster:ti,ab

#9 MeSH descriptor: [Varicellovirus] explode all trees

#10 MeSH descriptor: [Herpesvirus 3, Human] explode all trees

#11 HHV-3:ti,ab

#12 #1 OR #2 OR #3 OR #4 OR #5 OR #6 OR #7 OR #8 OR #9 OR #10 OR #11

#13 MeSH descriptor: [Medicine, Traditional] explode all trees

#14 alternative medicine*:ti,ab

#15 complementary medicine*:ti,ab

#16 MeSH descriptor: [Drugs, Chinese Herbal] explode all trees

#17 (plant OR plants):ti,ab

#18 MeSH descriptor: [Plant Extracts] explode all trees

#19 MeSH descriptor: [Plants, Medicinal] explode all trees

#20 MeSH descriptor: [Medicine, Kampo] explode all trees

#21 (ethnopharmacology OR ethnomedicine OR ethnobotany):ti,ab

#22 (TCM OR T.C.M.):ti,ab

#23 Herb*:ti,ab

#24 MeSH descriptor: [Herbal Medicine] explode all trees

#25 ((traditional OR chinese OR oriental OR herbal) near/2 medicine):ti,ab

#26 (phytodrug* OR phyto-drug* OR phytopharmaceutical*):ti,ab

#27 #13 OR #14 OR #15 OR #16 OR #17 OR #18 OR #19 OR #20 OR #21 OR #22 OR #23 OR #24 OR #25 OR #26

#28 #12 AND #27
